# Supplementary material for: Integrated laboratory protocol for the diagnosis of Sexually Transmitted Infections (STIs): Standardized pre-analytical procedures, rapid screening, hemagglutination, and ELISA methods for use in resource-limited settings
Source: PLoS One. 2026 May 5;21(5):e0346598. doi: 10.1371/journal.pone.0346598 (PMC13143095; doi:10.1371/journal.pone.0346598)
Supplement: S1 Table — (DOCX) [file pone.0346598.s001.docx]

**S1 Table . Performance characteristics of serological assays used in the integrated STI laboratory protocol.**

| Test | Sensitivity (%) | Specificity (%) | Source |
| --- | --- | --- | --- |
| HIV Rapid Immunochromatographic Test | 99–100 | ≥99 | Manufacturer data |
| Rapid Plasma Reagin (RPR) | 78–86 (primary infection) | 85–99 | Published literature |
| Treponema pallidum Hemagglutination Assay (TPHA) | >95 | >98 | Published literature |
| HBsAg ELISA | 98–100 | ≥99 | Manufacturer data |
| Anti‑HCV IgG ELISA | 97–99 | ≥99 | Manufacturer data |
| HSV‑2 IgG ELISA | 95–98 | 96–99 | Published literature |
| Chlamydia trachomatis IgG ELISA | 85–95 | 90–97 | Published literature |

Values represent approximate performance ranges reported in manufacturer documentation and previously published validation studies.

Abbreviations: ELISA, Enzyme‑Linked Immunosorbent Assay; RPR, Rapid Plasma Reagin; TPHA, Treponema pallidum Hemagglutination Assay; HSV‑2, Herpes Simplex Virus type 2.
