# Supplementary material for: Integrated laboratory protocol for the diagnosis of Sexually Transmitted Infections (STIs): Standardized pre-analytical procedures, rapid screening, hemagglutination, and ELISA methods for use in resource-limited settings
Source: PLoS One. 2026 May 5;21(5):e0346598. doi: 10.1371/journal.pone.0346598 (PMC13143095; doi:10.1371/journal.pone.0346598)
Supplement: S1 Checklist — (DOCX) [file pone.0346598.s004.docx]

**S1 Checklist. Lab Protocol Checklist**

*Manuscript: Integrated Laboratory Protocol for the Diagnosis of Sexually Transmitted Infections (STIs)*

**Purpose.** This checklist summarizes the key elements reported in the manuscript to facilitate editorial verification for the Lab Protocol submission.

| No. | Checklist item | Location in manuscript |
| --- | --- | --- |
| 1 | Protocol title clearly identifies the article as a laboratory protocol | Title page |
| 2 | Protocol DOI/link to protocols.io provided in the manuscript | Title page; Section 5.5 |
| 3 | Abstract summarizes the scope, assays, workflow, and intended use | Abstract |
| 4 | Scientific rationale and methodological justification are provided | Sections 2.1 to 2.3 |
| 5 | Objectives of the protocol are stated explicitly | Section 2.2 |
| 6 | Scope of use in resource-limited settings is defined | Sections 2.1 and 2.3 |
| 7 | Safety and biosafety requirements are described | Section 3.2 |
| 8 | Materials, equipment, reagents, and kits are specified | Section 4 |
| 9 | Pre-analytical procedures are described in detail | Section 5 |
| 10 | Sample transport and field constraints are addressed | Section 5.4 |
| 11 | Storage conditions and aliquoting procedures are described | Section 5.5 |
| 12 | Step-by-step analytical procedures are described | Section 6 |
| 13 | Interpretation guidance is provided for rapid tests, RPR, TPHA, and ELISA | Sections 6.1 to 6.4 |
| 14 | Quality assurance and quality control procedures are reported | Section 7 |
| 15 | Feasibility and cost considerations are discussed | Section 8 |
| 16 | Data management and traceability are described | Section 9 |
| 17 | Protocol limitations are discussed | Section 10 |
| 18 | Troubleshooting guidance is included | Section 11 |
| 19 | Workflow algorithm is summarized | Section 12 |
| 20 | Ethics statement is included in the manuscript | Ethics approval subsection |
| 21 | Supporting information captions are listed at the end of the manuscript | Supporting Information Captions |

**Note.** This checklist is provided as a supporting information file for editorial review. Please replace it with an official journal template if PLOS ONE specifically requests a different checklist format.
