# Supplementary material for: Integrated laboratory protocol for the diagnosis of Sexually Transmitted Infections (STIs): Standardized pre-analytical procedures, rapid screening, hemagglutination, and ELISA methods for use in resource-limited settings
Source: PLoS One. 2026 May 5;21(5):e0346598. doi: 10.1371/journal.pone.0346598 (PMC13143095; doi:10.1371/journal.pone.0346598)
Supplement: S1 File — (PDF) [file pone.0346598.s005.pdf]

Dec 08, 2025 Version 2

Integrated Laboratory Protocol for Diagnosis of Sexually TransmittedInfections (STIs): ELISA, Rapid Tests, Hemagglutination, and ConfirmatoryAlgorithms anti Treponema pallidum V.2

DOI
https://dx.doi.org/10.17504/protocols.io.261gek1wjg47/v2

Faramalala Ramamonjiarisoa, MD[1,2,3], HDR RAZAFIARIMANGA Zara Nomentsoa[4,5], Dr. Ramaroson Roseline PhD[6]

[1]Doctoral Candidate, Doctoral School of Life and Environmental Sciences (SVE);
[2]Research Team: EAD 7 – Immunology, Immunopathology, Immunodiagnostics;
[3]Medical Biologist, Research and Training Laboratory in Medical Biology (LBM), Faculty of Medicine, University of Antananarivo, Madagascar;
[4]Associate Professor – Director of Documentation, International Publications, and Editing, University of Antananarivo;
[5]President of the Faculty College of Teachers, Faculty of Sciences, University of Antananarivo;
[6]Specialization in Fundamental and Applied Biochemistry, Faculty of Sciences, University of Antananarivo, Madagascar

framamonjiarisoa

Create & collaborate more with a free account

Edit and publish protocols, collaborate in communities, share insights through comments, and track progress with run records.

Create free account

OPEN ACCESS

DOI: https://dx.doi.org/10.17504/protocols.io.261gek1wjg47/v2

Protocol Citation: Faramalala Ramamonjiarisoa, MD, HDR RAZAFIARIMANGA Zara Nomentsoa, Dr. Ramaroson Roseline PhD 2025. Integrated Laboratory Protocol for Diagnosis of Sexually TransmittedInfections (STIs): ELISA, Rapid Tests, Hemagglutination, and ConfirmatoryAlgorithms anti Treponema pallidum. **protocols.io** **https://dx.doi.org/10.17504/protocols.io.261gek1wjg47/v2**Version created by **framamonjiarisoa**

Manuscript citation:
Prevalence and Risk factors of Sexually Transmitted Infections (STIs) among malagasy youth

License: This is an open access protocol distributed under the terms of the **Creative Commons Attribution License**, which permits unrestricted use, distribution, and reproduction in any medium, provided the original author and source are credited

Protocol status: Working
We use this protocol and it's working

Created: December 05, 2025

Last Modified: December 08, 2025

Protocol Integer ID: 234256

Keywords: Treponema pallidum, CTK Biotech, Syphilis, HIV, RPR, TPHA, Rapid labs, ISO 15189, CLSI, WHO, Serological Testing, Diagnostic Algorithm, integrated diagnostic protocol for syphili, syphilis combo test for initial screening, syphilis combo test, tpha tests detection of antibody, standardized diagnostic workflow for syphili, syphilis combo, antibodies for both hiv, accurate syphili, treponema pallidum hemagglutination assay, syphili, confirming treponemal infection, using immunochromatographic hiv, hiv diagnosis, simultaneous hiv screening, tpha tests detection, immunochromatographic hiv, treponemal infection, specific antibody, syphilitic individual, treponema pallidum this protocol, antibody, hiv, antigen, serum bind to these antigen, treponema pallidum, specific antibodies in the plasma, rpr detects reagin, integrated diagnostic protocol, cholesterol particles in the test, diagnostic accuracy, overall diagnostic process, sexually transmittedinfection, treponemal assay, confirmatoryalgorithms anti treponema pallidum this

Abstract

This laboratory protocol describes a fully standardized and integrated diagnostic workflow for the detection of major sexually transmitted infections (STIs), including HIV-1/2, *Treponema pallidum*, Hepatitis B virus (HBV), Hepatitis C virus (HCV), *Chlamydia trachomatis*, and HSV-2. The workflow combines rapid immunochromatographic tests, non-treponemal and treponemal assays, hemagglutination testing, and ELISA-based antibody and antigen detection into a coherent algorithm.

The protocol is aligned with ISO 15189 and CLSI recommendations and is specifically designed to be feasible in resource-limited laboratories. It includes detailed pre-analytical requirements for blood collection, transport, and storage; standardized step-by-step procedures for each assay; internal and external quality assurance components; troubleshooting guidance; and recommendations for data management and sample traceability. This integrated approach aims to optimize diagnostic yield, ensure reproducibility, and support large-scale epidemiological studies or routine diagnostic activities in low-income settings where access to molecular testing is limited.

Attachments

protocole 05 DEC.pdf

395KB

Guidelines

All procedures were reviewed and approved by the Biomedical Ethics Committee of the University of Antananarivo. Written informed consent was obtained from adult participants, and assent with parental consent was required for minors prior to sample collection.

All serum handling must be performed in a Class II biological safety cabinet. Human specimens are considered potentially infectious and must be processed using universal safety precautions. Laboratory activities must comply with ISO 15190 standards for biosafety and facility protection.

Materials

Materials and Equipment

The following materials are required for blood collection: A 5mL venous blood sample collected into a dry Vacutainer tube with a red cap. Sterile single-use needles and compatible needle holders.
Pre-printed or on-demand barcoded labels for unique sample identification.
An insulated transport cool box (glacière) containing ice packs to maintain a temperature of approximately 2–8 °C during transport.

A refrigerated centrifuge capable of reaching 3000 rpm.
Adjustable micropipettes with appropriate disposable tips covering the relevantvolume ranges (e.g., 10–100 µL, 100–1000 µL).
An automated or manual ELISA microplate washer.
An ELISA microplate reader capable of reading absorbance at 450 nm, with optional reference wavelengths as recommended by the manufacturer.
Vortex mixer for homogenizing reagents and serum dilutions.
refrigerator maintained between 2–8 °C for short-term storage of reagents and specimens –80 C° ultra-low temperature freezer for long-term storage of serum aliquots.
At least one calibrated laboratory timer to monitor incubation and reaction steps.

The following reagents and diagnostic kits are required:
Combined rapid test kit for HIV and syphilis antibodies (e.g., CTK HIV/Syphilis Ab PLUS Combo), used for initial serological screening.

Non-treponemal rapid plasma reagin (RPR) test kit (e.g., BD Macro-Vue® RPR) for syphilis screening and titration.

Treponemal hemagglutination assay kit (e.g., TPHA Rapid Labs®) for syphilis confirmation.

Commercial ELISA kits for HBsAg, anti-HCV IgG, anti-HSV-2 gG-2 IgG, qualitative anti-*Chlamydia trachomatis* IgG, andHIV-1/2 as per manufacturer recommendations.

Tetramethylbenzidine (TMB) substrate solution for ELISA color development.

Ready-to-use or concentrated wash buffer (to be diluted to 1× as required) for microplate washing.

Manufacturer-supplied positive and negative controls and, when provided, calibrators for each ELISA kit and rapid test, stored and handled according to the instructions for use.

Troubleshooting

Problem

High background signal across many wells is most commonly associated with suboptimal washing, such as insufficient wash cycles, incomplete removal of residual liquid, or contamination of the wash buffer.

Solution

In such cases, the number of washing cycles should be increased, the washer function verified, and fresh wash buffer prepared if necessary.

Problem

Low signal for the positive control may indicate degraded or expired reagents, incorrect storage conditions, or deviations from the recommended incubation time or temperature.

Solution

When this occurs, reagent integrity and storage logs should be checked, incubation conditions reviewed, and, if required, a new kit lot used to repeat the assay.

Problem

High variability between duplicates (elevated CV) typically reflects pipetting inaccuracies or inconsistent timing.

Solution

This can be corrected by using calibrated pipettes, retraining staff in pipetting technique, and standardizing the order and timing of reagent addition.

Problem

Weak sample signals in the context of appropriate control performance may result from partial degradation of the specimen or repeated freeze thaw cycles. When feasible, testing should be repeated using a fresh or less-handled aliquot, and storage practices should be reviewed. If all wells show uniformly low signal, including controls, the most likely causes include omission of a key reagent such as the conjugate or substrate, incorrect sequence of steps, or major instrument malfunction.

Solution

In such situations, a full trace of the run should be performed, including verification of reagent addition, instrument logs, and staff training, before repeating the assay with newly prepared reagents.

Safety warnings

Perform procedures under Biosafety Level 2 (BSL-2) conditions. Wear gloves, laboratory coat, and protective eyewear. Dispose of waste according to local biosafety and environmental regulations. Treat all biological specimens as potentially infectious. Dispose of used materials in accordance with institutional biosafety and national biomedical waste guidelines. Sample Handling: Acceptable specimens: Serum or plasma (per kit IFU). Store samples at 2–8 °C up to 7 days or ≤ −20 °C for long-term storage. Avoid repeated freeze-thaw cycles.

Ethics statement

The study protocol was reviewed and approved by the Ethics Biomedical Committee of the University of Antananarivo. All procedures were conducted in accordance with national research ethics guidelines. Written informed consent was obtained from all participants. Calibrate pipettes and microplate readers. Store reagents at 2–8 °C and allow to reach room temperature before use. Verify internal quality controls (positive/negative sera) before performing

Before start

Pre-analytical Specifications

Venous blood is collected at a standardized volume of 5 mL into a dry serum tube without anticoagulant. Centrifugation is performed within a maximum of six hours after collection. Samples are transported to the laboratory in a cooler box containing refrigerated packs to maintain a stable temperature during transit. Serum intended for delayed testing is stored at −80 °C for long-term preservation, while short-term storage at +4 °C is acceptable for periods not exceeding seventy-two hours. When stored at −80 °C, serum samples maintain analytical integrity for several weeks before ELISA testing is performed.

Before You Begin

1 **Ethical approval**

All procedures described in this protocol were conducted in accordance with national and institutional ethical standards. The study was approved by the Biomedical Ethics Committee of the University of Antananarivo. Written informed consent was obtained from participants aged 18 years or older, and written informed assent was obtained from minors, accompanied by written consent from a parent or legal guardian, in line with national regulations governing research involving human participants. The protocol should not be implemented in new settings without prior approval from the relevant ethics committees and regulatory authorities.

2 **Safety considerations**

All human blood samples must be handled as potentially infectious regardless of the known
serostatus of the source patient. Work should be conducted in a laboratory that meets Biosafety Level 2 (BSL-2) requirements, with controlled access and appropriate biosafety infrastructure. Personnel must wear appropriate personal protective equipment, including laboratory coats, single-use gloves, and, when indicated, protective eyewear and surgical masks. Manipulations that may generate aerosols should be performed in a certified biological safety cabinet when available.
Hands must be washed or disinfected after glove removal and before leaving the laboratory.
Sharps (needles, lancets) should be disposed of immediately after use in puncture-resistant containers, and all biological waste must be inactivated and discarded in accordance with institutional biosafety procedures and national regulations. Spills of blood or serum should be treated with an appropriate
disinfectant, allowed sufficient contact time, and cleaned using absorbent material, with the event documented according to local biosafety guidelines. Regular training and refresher sessions in biosafety and waste management are strongly recommended for all staff involved in specimen handling and testing.

Pre-Analytical Procedures

3 Blood collection

3.1

Collect 5 mL of venous blood from each participant into a dry Vacutainer tube with a red cap using aseptic technique.

3.2 Immediately after collection, gently invert the tube three to five times to facilitate clot activation without causing hemolysis.

3.3 Affix a unique barcoded label to the tube and verify that the identifier matches the participant's study code or patient record.

3.4 Register the sample in the laboratory information management system (LIMS) or in a standardized paper log, recording the time of venipuncture and other relevant metadata.

4 Clotting and centrifugation

4.1 Allow the blood to clot in an upright position at room temperature for 30 to 60 minutes, away from direct sunlight or heat sources.

4.2 Centrifuge the clotted blood at 3000 rpm for 10 minutes to separate the serum from the cellular components.

4.3 5 Ensure that centrifugation is performed within six hours after venipuncture in order to minimize hemolysis and biochemical degradation.

5 Serum separation

5.1 After centrifugation, visually inspect the sample and carefully aspirate the clear supernatant serum using a pipette, avoiding transfer of red blood cells or buffy coat.

5.2 Transfer approximately 2–3 mL of serum into pre-labeled cryovials bearing the same unique identifier.

5.3 Check for evidence of gross hemolysis or lipemia and record this information, as it may influence assay performance and interpretation.

6 Transport

6.1 Place the cryovials containing serum in a sealed, leak-proof secondary container and then into an insulated transport cool box
.

6.2 Maintain a transport temperature between 2–8 °C and monitor, where possible, using a thermometer or temperature data logger.with ice packs
Ensure that the maximum transport time from the collection site to the testing laboratory does not exceed six hours.

7 Storage

7.1 For short-term storage of less than 48 hours before testing, keep serum samples at approximately +4 °C in a monitored refrigerator.

7.2 For long-term storage, as applied in this study, freeze serum aliquots at −80 °C.

7.3 Under these conditions, samples can generally be stored for several weeks before ELISA testing, provided that repeated freeze–thaw cycles are avoided by preparing multiple aliquots when possible.

Step 1 – Initial Rapid Screening (HIV/Syphilis Combo Rapid Test)

8 Bring the rapid test device and serum sample to room temperature if they have been refrigerated.
Apply 10 µL of serum to the sample well of the HIV/syphilis combo rapid test device using a calibrated pipette.
Add three drops of the manufacturer-supplied diluent to the appropriate well as indicated in the instructions for use.

9 Incubate the device at room temperature and read the result after 15 minutes, ensuring that the control line is present.
Interpretation:
6.1.5 If the HIV line is reactive, classify the sample as initially reactive for HIV antibodies and proceed to ELISA confirmation.
6.1.6 If the syphilis line is reactive, classify the sample as initially reactive for syphilis and proceed to non-treponemal RPR testing.

Step 2 – RPR (Non-Treponemal Test)

10 Place 50 µL of serum onto the designated circle of the RPR card using a pipette.
Add one drop of RPR antigen to the serum according to the manufacturer's guidelines.

11 Gently rotate the RPR card on a mechanical rotator for eight minutes at the recommended speed.

At the end of the rotation time, examine the mixture under adequate lighting to assess the presence or absence of flocculation.

12 Interpretation:
A reactive RPR result is characterized by visible flocculation and should be confirmed by a treponemal test such as TPHA.
In cases of high clinical suspicion with a non-reactive RPR, repeat testing or direct TPHA testing may be considered, following local guidelines.

6.3 Step 3 – TPHA (Treponemal Confirmation)

13 Dilute the serum sample at a ratio of 1:80 using the diluent provided with the TPHA kit.
Dispense 25 µL of the diluted serum into the appropriate test well of the microplate or reaction tray.

14 Add the sensitized erythrocytes (reagent cells) to the well according to the manufacturer's instructions.
Incubate the plate for 45 to 60 minutes at room temperature without disturbance.

15 Interpretation:
A diffuse layer of agglutinated cells covering the well surface is interpreted as a positive treponemal result.
A compact "button" of non-agglutinated cells at the bottom of the well is interpreted as a negative result.

6.4 Step 4 – ELISA Panel (HBsAg, Anti-HCV, Anti-HSV-2, Anti-Chlamydia trachomatis)

16 All ELISA kits are performed following a standardized procedure, while respecting manufacturer-specific details for each assay.
Sample preparation
Prepare a serum dilution at 1:21 by mixing 10 µL of serum with 200 µL of the assay diluent provided by the manufacturer.
Homogenize the mixture gently using a pipette or vortex mixer, avoiding the formation of bubbles.

16.1 ELISA procedure

Add 100 µL of the diluted serum sample into the designated wells of the microplate, according to the plate map.
Incubate the plate at 37 °C for 25 minutes to allow antigen–antibody binding.

Wash the plate for five cycles using an automated or manual washer, ensuring complete removal of residual liquid after each cycle.
Add 100 µL of conjugate solution to each well

16.2 Incubate the plate at 37 °C for an additional 25 minutes.
6.4.2.6 Repeat the washing step for five cycles to remove unbound conjugate.
6.4.2.7 Add 100 µL of TMB substrate to each well, protecting the plate from direct light during the color development step.

16.3 Incubate for 10 to 15 minutes at room temperature, monitoring the development of the color reaction if recommended.
Add 50 µL of stop solution to each well to terminate the reaction.
Read the absorbance at 450 nm within 30 minutes using the ELISA plate reader, applying any reference wavelength as indicated by the kit instructions

17 Interpretation (OD ratio index)

17.1

Calculate the optical density (OD) ratio or index according to the manufacturer's instructions, typically by comparing the sample OD to the cut-off control.

ratio or index of ≤ 0.90 is interpreted as negative.

17.2 A ratio or index between 0.91 and 1.09 is considered equivocal and may require repeat testing on the same or a new aliquot.
A ratio or index of ≥ 1.10 is interpreted as positive, and confirmatory testing should be performed when available.

17.3 Confirmatory testing:
For HIV, perform an immunoblot or nucleic acid test (NAT) according to local algorithms.
For HCV, confirm reactive results using HCV RNA testing.

17.4 For Chlamydia trachomatis, use NAAT or PCR when available to distinguish past exposure from active infection.
For HBsAg, perform a neutralization assay or an alternative confirmatory test as recommended.

Quality Assurance (QA/QC)

18 Internal controls

Each analytical run must include the full set of internal controls provided by the manufacturer. At a minimum, this includes one negative control, one positive control, and, where applicable, two or three calibrators that allow validation of the calibration curve or cut-off index. Controls should be treated in the same way as patient samples and placed on each plate in predefined positions to facilitate interpretation. Runs in which the controls do not meet the acceptance criteria must be invalidated and repeated.

19 External Quality Assessment

Participation in external quality assessment (EQA) or proficiency testing schemes is strongly recommended for HIV, HBV, and HCV serology, and for other analytes when available. EQA samples should be processed using the same procedures, reagents, and instruments as routine patient samples. Results must be reviewed by the laboratory supervisor, and any discrepancies or poor performance must trigger a documented investigation. Corrective and preventive actions should be recorded, implemented, and reviewed during periodic quality meetings.

20 7.3 Acceptance criteria

Assay validity is assessed against predefined acceptance criteria. For ELISA assays, the optical density of the negative control must remain below the threshold specified by the manufacturer, confirming low background. The positive control and calibrators must fall within the expected ranges or ratios, thereby validating the test run. Duplicate measurements of controls and samples should yield a coefficient of variation (CV) of less than 15 %, indicating acceptable repeatability. Any run failing these criteria requires identification of the cause (e.g., reagent degradation, instrument malfunction, washing errors) and repetition of the assay after corrective actions.

Data Management

21 All test results and associated metadata are recorded in a laboratory information management system (LIMS) or, when LIMS is not available, in a standardized and secure paper-based system. For each sample, the unique identifier, demographic information, collection date and time, processing details, test results, control performance, and operator identity are documented. Serum aliquots are cataloged and stored with clear location information (freezer, rack, and box position), and plate maps are archived to allow retrospective verification of results. Electronic records are backed up regularly, and access is restricted to authorized personnel only. Data confidentiality is ensured in accordance with national laws and institutional policies, including controlled access, password protection, and, where possible, de-identification of participant information for analytical purposes. Records related to testing and storage, including plate maps and

audit trails, are retained for at least two years or longer if required by national regulations or study protocols.

Limitations

22 This integrated serological protocol has several important limitations that must be considered during interpretation.
First, infections occurring during the early window period may not yet have induced detectable levels of antibodies or antigens, leading to false-negative results despite recent exposure.

23 Second, the Chlamydia trachomatis ELISA used in this protocol detects IgG antibodies indicative of past exposure and does not reliably distinguish between current active infection and resolved infection. In this protocol, Chlamydia trachomatis IgG ELISA is used solely as an epidemiological indicator of past exposure, consistent with international applications of CT serology in population-based surveillance programmes [1–5]. CT IgG assays cannot diagnose active infection, but they remain valuable for describing cumulative exposure among adolescents, especially where PCR is not feasible. Similarly, HSV-2 type-specific ELISA is included as a seroepidemiologic tool to quantify lifetime exposure at the population level, in line with global studies estimating HSV-2 burden across regions [6–9]. Both assays are therefore used here for public health surveillance rather than clinical decision-making.
ELISA IgG is not a diagnostic tool for detecting active Chlamydia trachomatis infection. We now explicitly state this in the manuscript. The purpose of including CT IgG ELISA in our protocol is strictly epidemiological. International literature demonstrates that CT serology particularly Pgp3- and OmcB-based assays is widely used to assess past exposure at the population level, monitor cumulative incidence, and support surveillance programmes when NAAT/PCR cannot be implemented at scale [1–5].
Large cohort studies and methodological reviews confirm that CT IgG assays provide valuable information on the historical burden of infection among adolescents and young adults, especially in low-resource settings where molecular testing is not feasible. We added these references and clarified in the text that CT serology is used only for seroepidemiological purposes and not for individual diagnosis.

24 For HBV and HCV, we implemented confirmation strategies aligned with WHO recommendations. HBsAg ELISA-reactive samples were confirmed using neutralization-based assays when resources allowed [10–12]. For HCV, RNA testing was performed where available, acknowledging that molecular confirmation remains limited in low-income laboratories and may affect diagnostic accuracy [10–13]. These constraints are explicitly recognized in the limitations section.
For HBV, we specify that:
Reactive HBsAg ELISA results were confirmed, when feasible, using a neutralization assay, following WHO and PAHO technical guidance [10–12]; neutralization-based

confirmation is validated as an appropriate and feasible strategy in low-resource laboratories, with strong performance documented in the literature [11–13].

25 For HCV, we now state explicitly that: HCV antibody positivity reflects exposure, not active infection;
definitive confirmation requires HCV RNA testing, according to WHO hepatitis testing guidelines [10];
RNA testing was performed when available, and the limitation of RNA access in resource-limited settings is now acknowledged.
We also added a section detailing expected performance constraints in low-resource environments, such as limited availability of neutralization reagents, automated MEIA/CLIA confirmation platforms, and molecular testing capacity [10–13].

26 Third , HSV-2 ELISA assays may demonstrate cross-reactivity with HSV-1 in some commercial kits, potentially reducing specificity. Finally, all initially reactive results obtained with screening ELISAs or rapid tests should be considered presumptive until confirmed by an appropriate reference or confirmatory test (e.g., immunoblot, neutralization, or nucleic acid testing), in line with local and international recommendations. We expanded the justification regarding HSV-2 type-specific ELISA. Type-specific gG-2 assays are internationally recognized as the reference method for HSV-2 seroprevalence studies and form the basis of global and regional HSV-2 burden estimates produced by WHO and international modelling groups [6–8]. These assays are specifically used to measure cumulative exposure, not to diagnose active genital herpes or guide clinical management at the individual level.
We now clearly state that HSV-2 ELISA in our protocol is intended exclusively for population-level surveillance, in line with current epidemiological practices [6–9], and does not inform treatment decisions.

27 Furthermore ,hemolysis, lipemia, or improper handling and storage of specimens can interfere with assay performance, leading to inaccurate optical density measurements or atypical control values.

Text-Based Workflow Algorithm

28 The overall diagnostic workflow begins with sample reception and verification, during which the identity of each specimen is checked against accompanying documentation and entered into the LIMS. Serum is then prepared through clotting, centrifugation, and aliquoting according to the pre-analytical procedures described above. All samples undergo initial rapid screening for HIV and syphilis using the combo rapid test.

29 Samples that are reactive for HIV on the rapid test proceed to ELISA confirmation, and, when available, to nucleic acid testing for definitive diagnosis and staging. Samples that

are reactive for syphilis on the rapid test undergo non-treponemal RPR testing, followed by treponemal TPHA confirmation when indicated. In parallel or subsequently, all serum samples are tested by ELISA for HBsAg, anti-HCV IgG, anti-HSV-2 IgG, and anti-Chlamydia trachomatis IgG. Reactive or equivocal ELISA results are followed by confirmatory or supplementary testing as available, such as NAT for HCV or NAAT for C. trachomatis.

30 At each step, internal quality controls are reviewed and external quality assessment performance is monitored. Only runs that meet predefined acceptance criteria are validated. Final results are compiled, verified by a qualified laboratory professional, and reported to clinicians or study investigators, with appropriate documentation of interpretation, limitations, and any additional recommendations for confirmatory testing or follow-up.

31 Sample Reception → Verification → Serum preparation → Rapid HIV/Syphilis screening →
If HIV reactive → ELISA confirmation → NAT if available → Report
If Syphilis reactive → RPR → TPHA → Report
ELISA panel: HBsAg, HCV IgG, HSV-2 IgG, CT IgG → Confirm where needed → QC →
Validation → Final report

Protocol references

1. Woodhall SC, et al. Advancing the public health applications of *Chlamydia trachomatis* serology. Lancet Infect Dis. 2018.
2. Waters MB, et al. *Chlamydia trachomatis* seroassays used in epidemiologic studies. 2024.
3.Hoenderboom BM, et al. Antibody testing to estimate past exposure to *Chlamydia trachomatis* in the Netherlands. Microorganisms. 2019.
4. Alexiou ZW, et al. Trends in *Chlamydia trachomatis* IgG seroprevalence in the Netherlands, 1996–2017. Sex Transm Infect. 2024.
5. Horner P, et al. Pgp3 antibody prevalence in young people. PLoS One. 2013.
6. Looker KJ, et al. Global estimates of HSV-2 infection burden in 2012. PLoS One. 2015.
7.Harfouche M, et al. Global incidence and prevalence of HSV-1/HSV-2. Sex Transm Infect. 2025.
8.Nasrallah GK, et al. Performance of four diagnostic assays for HSV-2 antibodies. J Clin Virol. 2019.
9. Abdo NM, et al. Seroprevalence of HSV-2 infection in fertility clinic patients. Front Public Health. 2022.
10. World Health Organization. Guidelines on Hepatitis B and C Testing. WHO; 2017.
11. Pan American Health Organization. Hepatitis B Surface Antigen Assays: Operational Characteristics. PAHO; 2001.
12. Abbott Diagnostics. HBsAg Confirmatory Reagent Kit – Package Insert. Abbott; 2019.
13. Fletcher GJ, et al. Neutralization assay for confirmation of HBsAg. J Gastroenterol Hepatol. 2010.
14. Han H, et al. Neutralization confirmatory testing for low-level HBsAg. 2024.
15. CTK Biotech. OnSite® HIV/Syphilis Ab Combo Rapid Test IFU
16. BD Diagnostics. Macro-Vue™ RPR Card Test IFU
17. Rapid Labs. TPHA 100–500T Rev 9, CE Single, 2023
18.WHO Laboratory Manual for STI Diagnosis, 2019
19. Larsen SA et al., J Clin Microbiol, 1981
20. Manufacturer Instructions for Use (IFU) for each specific kit
21. CLSI documents (e.g., EP05, EP12) for precision and qualitative test evaluation.
22.CDC. Sexually Transmitted Infections and Viral Hepatitis testing recommendations.

Acknowledgements

The authors thank the Research and Training Laboratory in Medical Biology (LBM), Faculty of Medicine, University of Antananarivo, and collaborating high schools. We also thank the Biomedical Ethics Committee and the participants for their cooperation.
