# Supplementary material for: Integrated laboratory protocol for the diagnosis of Sexually Transmitted Infections (STIs): Standardized pre-analytical procedures, rapid screening, hemagglutination, and ELISA methods for use in resource-limited settings
Source: PLoS One. 2026 May 5;21(5):e0346598. doi: 10.1371/journal.pone.0346598 (PMC13143095; doi:10.1371/journal.pone.0346598)
Supplement: S2 File — (DOCX) [file pone.0346598.s006.docx]

**S2 File Biosafety Notes – Safety and waste management**

These biosafety notes summarize minimum safety and waste-management practices for district- and regional-level laboratories.

- Biosafety level: Handle all human specimens as potentially infectious (universal precautions).
- PPE: Laboratory coat/gown, gloves, closed shoes; eye/face protection when splashes are possible.
- Engineering controls: Use a biosafety cabinet (Class II) for procedures with splash/aerosol risk (e.g., aliquoting, vortexing).
- Decontamination: Disinfect benches before/after work and after spills (e.g., freshly prepared 0.1–0.5% sodium hypochlorite, then 70% ethanol where appropriate).
- Sharps: Minimize use; dispose of needles/lancets immediately in puncture-resistant sharps containers.
- Waste segregation: Infectious waste in biohazard bags/containers; liquid waste treated with appropriate disinfectant before disposal per local regulations.
- Spill response: Cover spill with absorbent material, apply disinfectant, allow contact time, dispose as infectious waste, and document incident.
- Transport & storage: Leak-proof secondary containment; cold chain for reagents/specimens; clear labeling and logs.
- Staff training: Initial and periodic refresher training; incident reporting procedures; vaccination where indicated (e.g., HBV).
