# Supplementary material for: Integrated laboratory protocol for the diagnosis of Sexually Transmitted Infections (STIs): Standardized pre-analytical procedures, rapid screening, hemagglutination, and ELISA methods for use in resource-limited settings
Source: PLoS One. 2026 May 5;21(5):e0346598. doi: 10.1371/journal.pone.0346598 (PMC13143095; doi:10.1371/journal.pone.0346598)
Supplement: S3 File — (DOCX) [file pone.0346598.s007.docx]

**S3 Reagents and Equipment List – Integrated STI Diagnostic Protocol**

**S3 Table. Reagents and Equipment Used for the Integrated Diagnostic Protocol of Sexually Transmitted Viral Infections**

| **Category** | **Item** | **Manufacturer / Supplier** | **Catalog Number** | **Purpose** |
| --- | --- | --- | --- | --- |
| Sample Collection | Sterile vacutainer tubes (EDTA) | BD Biosciences | Ref TBD | Blood sample collection |
|  | Sterile disposable needles | Terumo | Ref TBD | Venipuncture |
|  | Alcohol swabs | Local medical supplier | NA | Skin disinfection |
|  | Disposable gloves | Medline | Ref TBD | Biosafety protection |
| Sample Processing | Centrifuge | Eppendorf | 5702 / equivalent | Plasma or serum separation |
|  | Micropipettes (0.1–1000 µL) | Eppendorf | Research Plus | Accurate liquid handling |
|  | Sterile pipette tips | Eppendorf | Ref TBD | Sample transfer |
|  | Cryovials (1.5–2 mL) | Nunc | Ref TBD | Sample storage |
| Serological Tests | HIV rapid test kits | Abbott / Determine | Ref TBD | Detection of HIV antibodies |
|  | HBsAg rapid test kits | SD Bioline / equivalent | Ref TBD | Detection of Hepatitis B surface antigen |
|  | Anti-HCV rapid test kits | SD Bioline / equivalent | Ref TBD | Detection of Hepatitis C antibodies |
|  | HSV-2 rapid test kits | Biokit / equivalent | Ref TBD | Detection of HSV-2 antibodies |
| Molecular Diagnostics (if applicable) | DNA/RNA extraction kit | Qiagen | Ref TBD | Viral nucleic acid extraction |
|  | PCR Master Mix | Thermo Fisher Scientific | Ref TBD | Amplification reactions |
|  | Specific primers | Custom synthesis | Ref TBD | Target gene amplification |
| Laboratory Equipment | Real-time PCR machine | Applied Biosystems | StepOnePlus / equivalent | Viral detection |
|  | Vortex mixer | Scientific Industries | Ref TBD | Sample homogenization |
|  | Biosafety cabinet (Class II) | Esco | Ref TBD | Safe sample handling |
|  | Refrigerator (4 °C) | Laboratory grade | NA | Reagent storage |
|  | Freezer (−20 °C / −80 °C) | Laboratory grade | NA | Long-term sample storage |
| General Laboratory Materials | Disposable lab coats | Local supplier | NA | Biosafety |
|  | Biohazard waste containers | Local supplier | NA | Safe disposal of contaminated materials |
|  | Disinfectant (70% ethanol or bleach solution) | Local supplier | NA | Surface decontamination |

**Abbreviations:**

HIV – Human Immunodeficiency Virus

HBsAg – Hepatitis B surface antigen

HCV – Hepatitis C Virus

HSV-2 – Herpes Simplex Virus type 2

*Note: Equivalent reagents and equipment with similar specifications may be used depending on laboratory availability.*
