# Supplementary material for: Integrated laboratory protocol for the diagnosis of Sexually Transmitted Infections (STIs): Standardized pre-analytical procedures, rapid screening, hemagglutination, and ELISA methods for use in resource-limited settings
Source: PLoS One. 2026 May 5;21(5):e0346598. doi: 10.1371/journal.pone.0346598 (PMC13143095; doi:10.1371/journal.pone.0346598)
Supplement: S4 File — (DOCX) [file pone.0346598.s008.docx]

# S4 Table Troubleshooting

**Troubleshooting guide (symptoms, causes, and corrective actions)**

| **Symptom / issue** | **Possible cause** | **Corrective action** |
| --- | --- | --- |
| High background OD in ELISA (all wells) | Insufficient washing; contaminated wash buffer; incorrect incubation temperature/time | Increase/verify wash cycles and soak time; prepare fresh 1× wash buffer; check washer function; confirm incubation settings and avoid plate drying between steps. |
| Low OD in positive controls | Reagents expired or stored incorrectly; wrong dilution; incorrect reader wavelength | Verify storage (2–8 °C) and expiry; remake dilutions; confirm reader set to 450 nm (and reference if required); repeat run with fresh controls. |
| Large variability between duplicate wells | Pipetting error; bubbles; uneven plate washing | Use calibrated pipettes; pre-wet tips; avoid bubbles; check washer dispense/aspirate uniformity; tap plate gently to remove bubbles before reading. |
| No agglutination in TPHA controls | Incorrect dilution; reagent not equilibrated; expired sensitized cells | Repeat dilution (1:80) carefully; bring reagents to room temperature as per IFU; replace kit if controls fail. |
| Weak/unclear RPR flocculation | Under-rotation/over-rotation; improper sample volume; temperature too low/high | Use recommended rotation speed/time; verify 50 µL volume; perform at recommended ambient temperature; repeat with fresh card/reagent. |
| Hemolysed sample affecting assay performance | Delayed centrifugation; transport temperature too high; rough handling | Prioritize centrifugation within 6–8 hours; transport at 2–8 °C; handle upright; reject/recollect if severe hemolysis and document pre-analytical deviation. |
| Rapid test invalid (no control line) | Insufficient sample/buffer; expired kit; incorrect procedure | Repeat with new device; verify sample/buffer volumes; check expiry and storage; retrain staff using SOP and job aids. |

*Note: If control criteria fail, do not report results; repeat the run after corrective actions and document deviations in the QC log.*
