# Supplementary material for: Integrated laboratory protocol for the diagnosis of Sexually Transmitted Infections (STIs): Standardized pre-analytical procedures, rapid screening, hemagglutination, and ELISA methods for use in resource-limited settings
Source: PLoS One. 2026 May 5;21(5):e0346598. doi: 10.1371/journal.pone.0346598 (PMC13143095; doi:10.1371/journal.pone.0346598)
Supplement: Attachment — Submitted filename: track changes 13 dec.pdf [file pone.0346598.s010.pdf]

Track change

~Strikethrough text~ = highlighted in yellow, content deleted on November 10

<<Added text>> = highlighted in blue, new content from December 05

(Comment: …) = justification when necessary to ensure compliance

Abstract

~Sexually transmitted infections (STIs) remain a significant public health concern in low- and middle-income countries, particularly in sub-Saharan Africa, where adolescents and young adults represent a highly vulnerable population. Early detection and treatment are essential to prevent long-term complications, transmission, and co-infection with HIV. In Madagascar, laboratory-based surveillance of STIs among youth remains limited due to financial and infrastructural constraints. This study was designed to develop and standardize an integrated diagnostic protocol combining rapid tests and enzyme-linked immunosorbent assays (ELISA) for accurate, reproducible, and context-appropriate detection of the major STIs affecting Malagasy adolescents.~

<<This laboratory protocol describes a fully standardized and integrated diagnostic workflow for the detection of major sexually transmitted infections (STIs), including HIV-1/2, Treponema pallidum, Hepatitis B virus (HBV), Hepatitis C virus (HCV), Chlamydia trachomatis, and HSV-2. The workflow combines rapid immunochromatographic tests, non-treponemal and treponemal assays, hemagglutination testing, and ELISA-based antibody and antigen detection into a coherent diagnostic algorithm.>>

<<The protocol is aligned with ISO 15189 and CLSI recommendations and is specifically designed to be feasible in resource-limited laboratories. It includes detailed pre-analytical requirements for blood collection, transport, and storage; standardized step-by-step procedures for each assay; internal and external quality assurance components; troubleshooting guidance; and recommendations for data management and sample traceability.>>

<<This integrated approach aims to optimize diagnostic yield, ensure reproducibility, and support large-scale epidemiological studies or routine diagnostic activities in low-income settings where access to molecular testing is limited.

The original text (November 10) focused on public health issues, the epidemiological context, and the justification of the protocol.

The revised version (December 05) completely removes this content and replaces it with:

- A technical, standardized description of the workflow
- ISO/CLSI alignment
- Detailed information on the tests included
- Positioning for use in resource-limited laboratories

INTRODUCTION

~Sexually transmitted infections remain a significant public health concern in low- and middle-income countries, particularly in sub-Saharan Africa, where adolescents and young adults
represent a highly vulnerable population. In many such contexts, access to molecular diagnostics
is restricted by cost, infrastructure, and human resource constraints. As a result, serological methods and rapid immunochromatographic tests constitute the main pillar of STI diagnosis in routine practice.~

<<Sexually transmitted infections remain a major public health challenge worldwide and are particularly difficult to manage in low-income and resource-limited settings.>>
(commentaire : Clarification of global vs contextual framing)

<<In many such contexts, access to molecular diagnostics is limited by cost, infrastructure, and
availability of trained personnel, making serological methods and rapid immunochromatographic
tests the primary tools for routine STI diagnosis.>>

(commentaire : Reformulation + precision on diagnostic constraints)
(Comment: Rephrasing and clarification of diagnostic constraints)

~This study was designed to develop and standardize an integrated diagnostic protocol combining
rapid tests and ELISA for accurate, reproducible, and context-appropriate detection of major STIs
affecting Malagasy adolescents.~

<<This protocol provides a harmonized, integrated laboratory workflow that was operationally
validated during a population-based study of adolescent STIs in Madagascar.>>
(commentaire : New emphasis on protocol validation)

<<By standardizing pre-analytical procedures, rapid screening, non-treponemal testing, treponemal confirmation, and ELISA testing for HIV, HBV, HCV, Chlamydia trachomatis, and
HSV-2, it offers a cost-effective and scalable diagnostic strategy.>>

<<The algorithm allows simultaneous or sequential testing for six key pathogens using equipment
and reagents that are commonly available in district or regional laboratories.>>

<<This integrated approach supports both clinical management and surveillance by improving consistency across sites, facilitating quality assurance, strengthening data comparability, and ensuring long-term sample storage for future analyses.>>

Deleted (November 10)

- Overly general public health context
- Emphasis on adolescent vulnerability
- References to epidemiology, not suitable for a laboratory-focused protocol
- Limited justification of the protocol

Added (December 05)

- Complete rewrite focused on:
- Standardization
- Operational validation
- Integration of methods (pre-analytical → testing → QA)
- Scalability in resource-limited laboratory settings
- Inter-site comparability

- Sample storage and traceability□ Supprimé (10 nov**)**
- Contexte trop généraliste de santé publique
- Mise en avant de la vulnérabilité des adolescents
- Références à l'épidémiologie, pas adaptées à un protocole *laboratory-focused*
- Justification légère du protocole

SECTION C — BEFORE YOU BEGIN

"BEFORE YOU BEGIN" — VERSION TRACK CHANGES

~Before starting, it is important to ensure that all reagents are available and that the laboratory technicians have been trained in the proper handling of STI diagnostic tests. Ethical approval should be obtained, and participants should be informed about the study objectives and procedures.~

<<Before performing the protocol, ensure that all ethical, biosafety, and pre-analytical requirements are met as described below.>>
(commentaire : Reformulation pour se conformer aux normes PLOS ONE)

ETHICAL APPROVAL

~Ethical approval should be obtained from relevant authorities, and informed consent must be collected from all participants or their guardians.~

<<This protocol was approved by the National Ethics Committee of Madagascar under ID CE-85/2018.>>
(commentaire : Précision obligatoire selon PLOS ONE)

<<Written informed consent (for adults) or parental consent with adolescent assent (for participants aged 14–17 years) was obtained before sample collection.>>

<<Specimen handling followed anonymization and data protection standards according to national guidelines.>>

SAFETY AND BIOSAFETY

~Technicians must wear gloves and handle all biological samples with care. Standard precautions should be followed.~

<<All procedures must be performed under standard biosafety level 2 (BSL-2) conditions.>>
<<Personal protective equipment (PPE) includes lab coat, gloves, closed shoes, and eye protection.>>
<<All biological waste must be discarded in accordance with national biosafety regulations and WHO infectious waste disposal recommendations.>>

PRE-ANALYTICAL REQUIREMENTS

~Ensure good sample quality and avoid hemolysis. Samples should be transported to the laboratory
as soon as possible.~

<<Collect 5 mL of venous blood in a dry tube. Avoid hemolysis and lipemia as these may affect
serological results.>>

<<Allow samples to clot at room temperature for 30–60 minutes, then centrifuge at 2500g for 10 minutes.>>
(commentaire : Standardisation ajoutée dans version 05 déc)

<<Serum must be aliquoted into cryovials and stored at 2–8°C for ≤72h or at -20°C for longer storage. Repeated freeze–thaw cycles must be avoided.>>

<<Label each aliquot with unique participant ID, date, sample type, and study code to ensure traceability within the LIMS system.>>

QUALITY CONTROL BEFORE TESTING

<<Verify expiration dates and storage conditions for all rapid tests, controls, calibrators, and ELISA reagents.>>

<<Run positive and negative controls with each batch. Deviations must be documented and investigated before results are validated.>>

SECTION D — MATERIALS (VERSION TRACK CHANGES)

REAGENTS

~Rapid diagnostic tests for HIV, syphilis, HBV, HCV, and other STI pathogens should be available. ELISA kits should also be prepared according to manufacturer recommendations.~

<<Prepare the following reagents before starting the protocol:>>

<<• HIV-1/2 Rapid Diagnostic Test (RDT), immunochromatographic format
(Manufacturer: SD Bioline or equivalent; Storage: 2–30°C)>>

<<• Syphilis rapid test (non-treponemal and treponemal formats)
RPR carbon antigen suspension (BD Diagnostics; Store: 2–8°C)
TPHA kit (Fujirebio; Store: 2–8°C)>>

<<• Hepatitis B surface antigen (HBsAg) RDT (e.g., Determine™ HBsAg; Store: 2–30°C)>>

<<• HCV antibody rapid test (e.g., SD Bioline Anti-HCV; Store: 2–30°C)>>

<<• ELISA kits for:
– HIV Ag/Ab Combo (e.g., Vironostika, Ref. #xxx; 2–8°C)
– HBsAg ELISA (Ref. #xxx; 2–8°C)
– Anti-HCV IgG ELISA (Ref. #xxx; 2–8°C)
– Chlamydia trachomatis IgG ELISA (NovaTec, Ref. #xxx; 2–8°C)
– HSV-2 IgG ELISA (Euroimmun, Ref. #xxx; 2–8°C)>>

(commentaire : Tous ces kits sont explicitement listés dans la version révisée pour conformité reproductibilité.)
(Comment: All these kits are explicitly listed in the revised version to ensure reproducibility compliance.)

CONTROLS & CALIBRATORS

~Positive and negative controls should be included.~

<<Include internal controls provided with each ELISA kit (positive control, negative control, cut-off calibrator).>>

<<Prepare external quality controls (EQA) when available. Note storage conditions (typically -20°C).>>

CONSUMABLES

~Gloves, pipettes, tubes, and alcohol wipes are needed.~

<<• Venous blood collection materials (21G needles, vacutainers, dry tubes)>>
<<• Cryovials (1.5–2 mL) for aliquots>>
<<• Micropipette tips (10 µL, 200 µL, 1000 µL), sterile and filtered>>
<<• ELISA microplates (included in kits)>>
<<• Serological pipettes and reservoirs>>
<<• Absorbent tissues, disinfectants (70% ethanol, bleach)>>
<<• Timer and tube racks>>

(Comment: Detailed list.)

EQUIPMENT

~A centrifuge and ELISA reader are required.~

<<The following equipment is required:>>
<<• Refrigerated centrifuge (capable of 2500–3000g)>>
<<• ELISA microplate reader (450 nm primary filter; 620–630 nm reference filter)>>
<<• Incubator (37°C)>>
<<• Refrigerator (2–8°C) and freezer (-20°C)>>
<<• Class II biosafety cabinet (recommended)>>
<<• Laboratory timer>>
<<• Computer with LIMS or data-entry software>>

STORAGE CONDITIONS

~Reagents should be stored properly.~

<<Store all ELISA reagents at 2–8°C. Do NOT freeze.>>
<<Rapid tests may be stored at 2–30°C unless stated otherwise.>>
<<Serum samples must be processed within 72h at 2–8°C or frozen at -20°C for long-term storage.>>
<<Record all storage conditions in laboratory logs for QA purposes.>>

DOCUMENTATION

<<Prepare laboratory notebooks, result sheets, and LIMS access.>>
<<Maintain traceability through unique sample identifiers (ID codes).>>

E1 — LABORATORY PREPARATION

~Ensure that the laboratory is clean and that equipment is functioning. Prepare all reagents according to manufacturer instructions.~

<<Before initiating the protocol, perform a full laboratory readiness check as described below.>>

<<• Verify that the laboratory temperature (20–25°C) and humidity are within acceptable ranges.>>
(commentaire : ajout pour conformité ISO 15189)

<<• Confirm calibration status of pipettes, centrifuge, and ELISA reader. Document in QA logbook.>>

<<• Prepare biosafety cabinet (BSL-2) and disinfect all surfaces with 70% ethanol.>>

<<• Lay out all rapid tests, ELISA plates, controls, and consumables on a clean, dry, level surface. Do NOT open foil packages until immediately before use.>>

<<• Ensure uninterrupted power supply for incubators, timers, and microplate reader.>>

□ E2 — SAMPLE COLLECTION AND PROCESSING (TRACK CHANGES)

~Collect blood samples and allow them to clot before performing the tests. Hemolyzed samples
should be avoided.~

<<Collect 5 mL of venous blood in a dry tube using aseptic technique.>>

<<Label each tube with participant ID, date, and sample type. Confirm identity before collection
to avoid mismatches.>>

<<Allow the sample to clot upright for 30–60 minutes at room temperature.>>

<<Centrifuge samples at 2500g for 10 minutes to separate serum.>>

<<Aliquot serum into sterile cryovials (1.5–2 mL), ensuring at least 500 µL per aliquot for multiple assays.>>

<<Store serum as follows:
– 2–8°C for ≤72h
– -20°C for longer storage (weeks to months)
Avoid repeated freeze–thaw cycles.>>

<<Record all steps in the LIMS system, including time of collection, processing time, and storage
location.>>

~Proceed directly to testing whenever possible.~

<<If testing cannot be performed the same day, store serum appropriately as indicated above.>>

E3 — HIV RAPID DIAGNOSTIC TEST (TRACK CHANGES)

~Use an HIV rapid test according to the manufacturer's instructions. Add the required sample volume and buffer, then read after the recommended time.~

<<Perform HIV-1/2 screening using a WHO prequalified immunochromatographic rapid diagnostic
test (RDT).>>

<<1. Allow test device, serum samples, and buffer to reach room temperature (20–25°C).>>
(commentaire : conformités pré-analytiques)

<<2. Open foil pouch immediately before use. Do NOT use if desiccant is pink (humidity failure).>>

<<3. Add 50–100 µL of serum to the sample well using a calibrated micropipette.>>

<<4. Add 1–2 drops (≈ 60–80 µL) of assay buffer to the buffer well.>>

<<5. Start timer immediately. Incubate for 15 minutes (do not read before 15 min; do not read after 20 min).>>

~Interpret the result visually.~
<<6. Interpret results as follows:
– Two lines (test + control) = HIV reactive
– One control line only = HIV non-reactive
– No control line = Invalid; repeat the test>>

<<7. Document lot number, expiry date, and operator initials in QA logbook.>>

E4 — SYPHILIS RAPID TEST (TREPO + NON-TREPO) — TRACK CHANGES

(A) Rapid Screen for Treponemal Antibodies

~Syphilis rapid tests can be used to detect Treponema pallidum antibodies.~

<<Use a treponemal-specific rapid diagnostic test for initial syphilis screening.>>

<<1. Bring test cassette, buffer, and serum to room temperature.>>
<<2. Add 50 µL serum to sample well.>>
<<3. Add 1 drop of buffer.>>
<<4. Incubate 10–15 min.>>

<<Interpretation:
– Two lines = Treponemal antibody reactive
– One control line = Non-reactive
– No control line = Invalid>>

(B) NON-TREPONEMAL TEST — RPR (SECTION E5)

~RPR testing is used to detect non-treponemal antibodies. Rotate the card and read the result.~

<<Use a carbon antigen-based RPR test to detect non-treponemal antibodies.>>

<<1. Prepare RPR card on a flat, vibration-free surface.>>
<<2. Pipette 50 µL of serum into each circular test field.>>
<<3. Resuspend antigen by gentle inversion; add 1 drop (≈ 20–30 µL) to each field.>>
<<4. Place card on a rotator at 100 RPM for 8 minutes.>>

~Read results visually.~
<<5. Immediately examine for flocculation (reactive vs non-reactive).>>

<<6. Semi-quantitative titration (if needed): Perform serial 1:2 dilutions until endpoint.>>

<<7. Record titer and reagent lot number (required for QA).>>

E6 — TREPONEMAL CONFIRMATION — TPHA (TRACK CHANGES)

~TPHA can be used to confirm syphilis infection when available.~

<<Perform Treponema pallidum hemagglutination assay (TPHA) for confirmation of treponemal
antibodies.>>

<<1. Prepare V- or U-bottom microplate.>>
<<2. Add 25 µL serum to designated well; dilute 1:80 or per manufacturer recommendations.>>
<<3. Add equal volume of sensitized red blood cells.>>

<<4. Gently tap to mix; avoid bubbles.>>
<<5. Incubate at room temperature for 45–60 minutes without disturbance.>>

~Interpret results as reactive or non-reactive.~
<<Interpretation:
– Smooth “carpet” pattern = Positive TPHA
– Tight “button” pattern = Negative
– Mixed patterns = Indeterminate; repeat test>>

SECTION E7 — HBV & HCV RAPID TESTS (TRACK CHANGES)

~HBV and HCV can be screened using rapid tests according to manufacturer instructions.~

<<Perform HBV and HCV screening using WHO prequalified immunochromatographic rapid tests.>>

HBsAg Rapid Test

<<1. Allow test cassette and serum to reach room temperature (20–25°C).>>
<<2. Add 60–80 µL of serum into the sample well.>>
~Add sample and wait for the result.~
<<3. Add 1–2 drops of buffer (if applicable).>>
<<4. Incubate for 15 minutes. Do NOT read after 20 minutes.>>

<<Interpretation:
– Two lines = HBsAg reactive
– One control line = Non-reactive
– No control line = INVALID; repeat using new cassette>>

HCV Rapid Test

~The anti-HCV rapid test is performed similarly.~
<<Follow the same procedure as above with manufacturer-specific volumes.>>

<<Interpretation is analogous:
– Two lines = Anti-HCV antibody reactive
– One control line = Non-reactive
– No control line = Invalid>>

□ SECTION E8 — HIV Ag/Ab ELISA

~Perform the HIV ELISA according to manufacturer instructions. Add sample, incubate, wash, and read.~

<<Use a fourth-generation HIV Ag/Ab ELISA kit. All steps must follow ISO 15189-compliant
procedures.>>

Plate Preparation

<<1. Bring all reagents to room temperature (20–25°C) for 30 minutes.>>
<<2. Do NOT thaw calibrators or controls more than once.>>
<<3. Allocate wells for NC, PC, calibrator, and samples (run in duplicates).>>

Assay Procedure

<<4. Add 100 µL of sample, NC, PC, and calibrator into designated wells.>>
<<5. Cover plate and incubate at 37°C for 30 minutes.>>

~Wash the plate.~
<<6. Wash plate 5× with 300 µL/well wash buffer using automated washer. Ensure no residual liquid remains.>>

<<7. Add 100 µL conjugate solution.>>
<<8. Incubate at 37°C for 30 minutes.>>
<<9. Repeat washing step.>>
<<10. Add 100 µL TMB substrate. Incubate 10–15 minutes in the dark.>>

<<11. Add 50 µL stop solution. Mix gently without splashing.>>

Reading & Interpretation

<<12. Read optical density at 450 nm with 620–630 nm reference.>>

~Interpret positive or negative.~
<<13. Calculate cut-off index (COI) per manufacturer formula.>>

<<14. Interpretation:
  – COI ≥ 1.0 = Reactive
  – COI < 1.0 = Non-reactive>>

<<15. Repeat testing for borderline COI (e.g., 0.9–1.1).>>

□ SECTION E9 — HBsAg ELISA (TRACK CHANGES)

~HBsAg ELISA follows standard ELISA procedures.~

<<Perform HBsAg ELISA using kit-specific instructions with standardized steps below.>>

Procedure

<<1. Add 100 µL serum to wells.>>
<<2. Incubate at 37°C for 60 minutes.>>
<<3. Wash plate 5×.>>
<<4. Add 100 µL enzyme conjugate. Incubate 30 minutes at 37°C.>>
<<5. Wash 5× again.>>
<<6. Add 100 µL TMB substrate, incubate 15 minutes in dark.>>
<<7. Add 50 µL stop solution.>>

Interpretation

<<Read OD at 450 nm (reference 630 nm).>>

~Interpret as per kit.~
<<Interpretation:
  – Sample OD ≥ Cut-off = Positive for HBsAg
  – Sample OD < Cut-off = Negative>>

SECTION E10 — Anti-HCV IgG ELISA (TRACK CHANGES)

~Perform Anti-HCV ELISA by adding sample and incubating.~

<<Use anti-HCV IgG ELISA with the following standardized workflow.>>

<<1. Dispense 100 µL sample into wells.>>
<<2. Incubate 60 minutes at 37°C.>>
<<3. Wash plate 5×.>>
<<4. Add 100 µL conjugate; incubate 30 minutes.>>
<<5. Wash 5×.>>
<<6. Add substrate; incubate 10–15 minutes.>>
<<7. Add stop solution.>>

<<Interpretation follows manufacturer-specific COI thresholds.>>

SECTION E11 — Chlamydia trachomatis IgG ELISA (TRACK CHANGES)

~CT IgG ELISA detects prior exposure.~

<<Perform CT IgG ELISA following NovaTec kit instructions with standardized steps.>>

<<1. Add 100 µL diluted sample (1:100 dilution recommended).>>

<<2. Incubate for 60 minutes at 37°C.>>
<<3. Wash plate 5×.>>
<<4. Add conjugate; incubate 30 minutes.>>
<<5. Wash 5×.>>
<<6. Add TMB substrate; incubate 15 minutes.>>
<<7. Add stop solution.>>

<<Interpret via NTU (NovaTec Units):
– NTU < 9 = Negative
– 9–11 = Borderline
– > 11 = Positive>>

□ SECTION E12 — HSV-2 IgG ELISA (TRACK CHANGES)

~HSV ELISA can be used to detect HSV infection.~

<<Perform HSV-2 IgG ELISA using Euroimmun kit.>>

<<1. Dilute serum 1:101 in sample buffer.>>
<<2. Add 100 µL to wells. Incubate 60 minutes at 37°C.>>
<<3. Wash 3–5× depending on washer.>>
<<4. Add enzyme conjugate; incubate 30 minutes.>>
<<5. Wash plate.>>
<<6. Add substrate; incubate 15 minutes in dark.>>
<<7. Add stop solution.>>

<<Interpretation based on ratio (sample OD ÷ calibrator OD):
– Ratio < 0.8 = Negative
– 0.8–1.1 = Borderline
– > 1.1 = Positive>>

□ SECTION E13 — QUALITY CONTROL, READING & RESULT ACCEPTANCE

~Ensure that controls give expected results. Repeat tests if needed.~

<<Perform internal quality control (QC) as required for each batch of tests and ELISA plate.>>

RAPID TEST QC

<<1. Check whether the internal control line appears on each rapid test. Tests without control line MUST be discarded and repeated.>>

<<2. Document lot numbers, expiry dates, and operator identity in QC logbook.>>

ELISA QC

~Ensure plate reading is correct.~
<<3. Verify that PC, NC, and calibrator fall within manufacturer-defined ranges.>>

<<4. Plate acceptance criteria (example):
– NC OD ≤ 0.150
– PC OD ≥ manufacturer threshold
– Calibrator OD within acceptable interval>>

<<5. Reject entire plate if NC fails, if PC is below threshold, or if calibrator is out-of-range.>>

READING OF RESULTS

~Read OD at 450 nm.~
<<6. Read optical density (OD) at 450 nm with 620–630 nm reference filter.>>

<<7. Duplicate wells must have coefficient of variation (CV%) < 15%.>>
(commentaire : Ajout requis par PLOS One et ISO 15189)

<<8. Re-run samples with discrepant duplicates.>>

RESULT ACCEPTANCE

<<9. Results are accepted only if all QC elements pass.>>

<<10. Record all QC outcomes in laboratory logbook and LIMS.>>

□ SECTION E14 — RESULT VALIDATION & NON-CONFORMITY MANAGEMENT

~Validate results after checking controls.~

<<Validation of results must follow ISO 15189 and laboratory SOP requirements.>>

PRIMARY VALIDATION

<<1. The laboratory analyst verifies:
– QC acceptability
– duplicate agreement

– correct sample identifiers
– absence of technical errors during testing>>

<<2. Any doubtful or borderline sample must be retested in duplicate.>>

SECONDARY VALIDATION

<<3. Senior laboratory supervisor reviews results, signs validation sheet, and approves data release.>>

NON-CONFORMITY MANAGEMENT

~Repeat tests if something is wrong.~

<<4. Document any deviation, interruption, or improper reagent handling in the Non-Conformity
(NC) register.>>

<<5. Investigate root cause (equipment, operator, reagents, environment).>>

<<6. Perform corrective action and preventive action (CAPA) and document in CAPA logbook.>>

<<7. If results are invalid or QC fails, testing must be repeated with new reagents or new sample
aliquots.>>

□ SECTION E15 — TROUBLESHOOTING

(VERSION TRACK CHANGES — intégré selon modèle PLOS ONE)

~If tests do not work, verify procedure.~

<<Common troubleshooting scenarios are provided below.>>

SCENARIO 1 — HIGH BACKGROUND IN ELISA

~Check washing.~
<<Cause: Insufficient washing or contaminated wash buffer.>>
<<Solution: Increase wash cycles; prepare fresh wash buffer.>>

SCENARIO 2 — LOW PC OD

<<Cause: Degraded positive control, incorrect incubation temperature, expired reagents.>>
<<Solution: Prepare new controls; verify incubator temperature; replace reagents.>>

SCENARIO 3 — HIGH CV% BETWEEN DUPLICATES

<<Cause: Pipetting inconsistency or presence of bubbles.>>
<<Solution: Re-pipette, avoid bubbles, use calibrated pipettes.>>

SCENARIO 4 — EDGE EFFECT IN ELISA PLATE

<<Cause: Uneven incubation or evaporation.>>
<<Solution: Use plate seals; incubate uniformly; avoid outer wells if possible.>>

SCENARIO 5 — INVALID RAPID TESTS (NO CONTROL LINE)

<<Cause: Humidity exposure or expired cassette.>>
<<Solution: Repeat with new cassette; store tests properly.>>

□ SECTION F — EXPECTED OUTCOMES (TRACK CHANGES)

~This protocol enables the diagnosis of STIs and provides results for epidemiological studies.~

<<Successful implementation of this protocol yields reproducible, high-quality serological and
rapid-test results for six major STIs: HIV, Treponema pallidum, HBV, HCV, Chlamydia trachomatis,
and HSV-2.>>

<<Expected outcomes include:>>

<<• Clean and interpretable rapid test bands for HIV, syphilis, HBV, and HCV.>>
<<• ELISA plates with low background signal, acceptable NC and PC values, and CV% < 15%.>>
<<• Consistent cut-off index (COI), NTU, or ratio-based interpretation depending on assay.>>
<<• High-throughput processing suitable for large-scale epidemiological studies.>>
<<• Traceable sample records in LIMS allowing longitudinal follow-up or re-testing.>>

~The protocol provides basic results.~

<<This protocol ensures standardized, comparable results across laboratories in resource-limited settings.>>

□ SECTION G — LIMITATIONS (TRACK CHANGES)

~The main limitation is that ELISA cannot detect acute infection in the window period.~

<<Limitations of this integrated protocol must be acknowledged to avoid misinterpretation:>>

<<1. Serology-based assays cannot reliably detect acute infections occurring within early window periods, potentially leading to false-negative results.>>

~Chlamydia ELISA is not very specific.~
<<2. Chlamydia trachomatis IgG ELISA detects past exposure, not active infection, which may limit its utility for clinical diagnosis but remains valuable for seroepidemiology.>>

<<3. HSV-2 serology may show cross-reactivity or fail to differentiate between primary and recurrent infection.>>

<<4. Rapid tests may exhibit reduced sensitivity in low-prevalence populations.>>

<<5. The protocol does not include molecular confirmation due to resource constraints; NAAT remains the gold standard when available.>>

<<6. Cold-chain interruptions or improper reagent handling may affect assay performance.>>

□ SECTION H — SAFETY STATEMENT (TRACK CHANGES)

~Handle all samples with care and wear gloves.~

<<All procedures must comply with biosafety level 2 (BSL-2) requirements.>>

<<Personnel must wear PPE including gloves, lab coat, and eye protection.>>

<<All blood-derived materials must be considered potentially infectious.>>
<<Avoid needle-stick injuries; dispose of sharps in approved containers.>>

<<Decontaminate surfaces with 70% ethanol or 0.5% sodium hypochlorite.>>

<<Waste disposal must follow national biosafety regulations and WHO recommendations.>>

<<All incidents, exposures, or spills must be documented and reported to the biosafety officer.>>

SECTION I — REFERENCES

Important note:

Comparison of the files shows that:

The November 10 version had an incomplete reference list, with some duplicated text.

The December 05 version removed duplicates, reorganized the references, and added missing ones, in accordance with PLOS ONE editorial recommendations (which had flagged repetitions).

~References were listed here but included duplicated entries and lacked standard formatting.~

<<References have been reorganized according to PLOS ONE citation style.>>

<<Duplicate entries have been removed.>>

<<New references have been added to document:
– ELISA procedures
– QC and ISO 15189 guidelines
– WHO STI testing recommendations
– Rapid test validation studies>>

<<All citations now follow numerical order of appearance.>>
