# Supplementary material for: Integrated laboratory protocol for the diagnosis of Sexually Transmitted Infections (STIs): Standardized pre-analytical procedures, rapid screening, hemagglutination, and ELISA methods for use in resource-limited settings
Source: PLoS One. 2026 May 5;21(5):e0346598. doi: 10.1371/journal.pone.0346598 (PMC13143095; doi:10.1371/journal.pone.0346598)
Supplement: Attachment — Submitted filename: PONE-D-25-65592_my comments.pdf [file pone.0346598.s012.pdf]

Integrated Laboratory Protocol for the Diagnosis of Sexually Transmitted Infections (STIs): Standardized Pre-Analytical Procedures, Rapid Screening, Hemagglutination, and ELISA Methods for Use in Resource-Limited Settings

Thank you for submitting the manuscript; it's very relevant in LMIC and resource-constrained settings.

Strengths

1. It addresses a critical gap by providing a standardized workflow for the 6 STIs common in LMICs.
2. The processes were aligned with standards like ISO 15189, CLSI, and WHO guidelines.
3. The manuscript clearly distinguishes between tests used for clinical diagnosis (HIV, HBV, Syphilis) and those used strictly for seroepidemiological assessment (Chlamydia, HSV-2, HCV).

Areas of improvement

1. There seems to be an assumption that at the sample collection site, there will be a mini laboratory where serum separation will occur. This will not be applicable in all settings. So please provide guidelines for when samples are collected in the field and then transported to the laboratory.
2. It was specified in section 6.4.2 that ELISA incubation time (37 °C for 25 minutes) and washing cycles (5 cycles) for all assays in the panel. While this simplifies the workflow, it may conflict with specific manufacturer instructions for different commercial kits. Please clarify if these specific parameters were validated across multiple brands or if they are specific to the kits used in your study.
3. The troubleshooting section should be more robust – include a table of specific "Troubleshooting symptoms, possible causes, and corrective actions" to assist laboratory technicians in real-time.
4. You mentioned that "The required equipment is compatible with the capacity of district-level laboratories, and the storage and transport conditions of the reagents are well adapted to Malagasy field settings". Best to define what Malagasy field settings mean in practical terms – environmental conditions, etc. This might help if your method is to be adapted to other settings.
5. You asserted that the protocol is a "cost-effective and scalable diagnostic strategy" but the manuscript lacks a basic comparative cost analysis (e.g. estimated cost per

sample for this integrated panel versus individual molecular tests). Can you provide a range of expected costs to strengthen the manuscript or re-word your assertions?

6. There are several minor errors, such as "optique density" instead of "optical density," "approuved" instead of "approved," and "Commitée" instead of "Committee.
7. While you have stated clearly that some of the STI results cannot be used for clinical assessment, e.g. Chlamydia, how does a clinician in this setting manage such cases? Advise how the diagnosis of active infection can be integrated with your protocol.

Thank you for the manuscript.
