# Supplementary material for: Integrated laboratory protocol for the diagnosis of Sexually Transmitted Infections (STIs): Standardized pre-analytical procedures, rapid screening, hemagglutination, and ELISA methods for use in resource-limited settings
Source: PLoS One. 2026 May 5;21(5):e0346598. doi: 10.1371/journal.pone.0346598 (PMC13143095; doi:10.1371/journal.pone.0346598)
Supplement: Attachment — Submitted filename: Plos one review.docx [file pone.0346598.s011.docx]

Dear editor and authors

The manuscript defines a lab protocol provides a harmonized, integrated laboratory workflow that was operationally validated during a population-based study of adolescent STIs in Madagascar.

This protocol offers a validated, integrated laboratory workflow combining: rapid immunochromatographic screening, non-treponemal and treponemal confirmatory testing, hemagglutination assays,and multi-pathogen ELISA panels (HBsAg, HCV IgG, HSV2 IgG, CT IgG), with pre-analytical guidance, QC requirements, and confirmatory pathways adapted to LMICs. The protocol is distinct from routine clinical algorithms because It has been operationally validated in an adolescent population.

The manuscript and lab protocol was well defined and written however I have some minor suggestions;

1- HSV2 IgG and CT IgG positivity can be used to assess the seroprevalence of past infections, not for diagnostic purposes. As the authors point out as a limitation, it is not meaningful for active infection screening without an IgM result. It can be used for surveillance purpose.
2- The authors state that the protocol offers a cost-effective and scalable diagnostic strategy. However they do not give any cost effectivenes data. Please add if there is any data on it.
3- The protocol was well defined but I think it is important to give the sensitivity and specifity of the test to predict false negatives and positives results.
4- Abbreviations such as QC, SOPs etc. should be given in their full form the first time they appear in the manuscript.
5- The entire article should be reviewed for spelling errors and any typos should be corrected. Punctuation marks should be added without leaving a space after the word, and then a space should follow. For example ‘Keysword :’ should be written as ‘Keywords:’ , ‘DOI :’ as ‘DOI:’ etc.
