# Supplementary material for: Integrated laboratory protocol for the diagnosis of Sexually Transmitted Infections (STIs): Standardized pre-analytical procedures, rapid screening, hemagglutination, and ELISA methods for use in resource-limited settings
Source: PLoS One. 2026 May 5;21(5):e0346598. doi: 10.1371/journal.pone.0346598 (PMC13143095; doi:10.1371/journal.pone.0346598)
Supplement: Attachment — Submitted filename: PONE-D-25-65592R1_Revised_Response.docx [file pone.0346598.s013.docx]

**Response to PLOS ONE Submission Check (PONE-D-25-65592R1)**

Dear PLOS ONE Editorial Office,

Thank you for checking our submission. We have addressed the requested items as follows:

**1) protocols.io PDF and DOI:**

We will upload the protocols.io-generated PDF of the step-by-step protocol as Supporting Information titled “S1 File” (filename: S1_file.pdf) and provide the protocols.io DOI in the submission form. We added the required sentence in the Methods section of the manuscript: “The protocol described in this peer-reviewed article is published on protocols.io (https://dx.doi.org/10.17504/protocols.io.261gek1wjg47/v2) and is included for printing purposes as S1 File.”.

**2) Ethics statement location:**

We ensured that the ethics statement appears only in the Methods section. The ethics text previously included in “Before You Begin” has been removed from that section and placed in Methods under an “Ethics approval” subsection.

**3) Supporting Information files cited in the manuscript:**

We will upload the Supporting Information files referenced in the manuscript. To comply with “S1 File” being reserved for the protocols.io PDF, Supporting Information has been renumbered and all in-text citations updated accordingly: S2 Flowchart (Workflow diagram), S3 Biosafety Notes (Safety and waste management), S4 Ethics Statement (Ethics approval & anonymization), S5 Dataset (QC and technical data), S6 Checklist, S7 Troubleshooting Table, S8 Reagents List, S9 Table (assay performance) and S10 Dataset

Supporting Information captions have been formatted according to PLOS ONE guidance.

Sincerely,

Faramalala Mamitiana Ramamonjiarisoa
